# Supplementary material for: From sequence to enzyme mechanism using multi-label machine learning
Source: BMC Bioinformatics. 2014 May 19;15:150. doi: 10.1186/1471-2105-15-150 (PMC4229970; doi:10.1186/1471-2105-15-150)
Supplement: Additional file 2 — Java code of ml2db. Additional file ml2db_code.tar.gz contains the Java source code to run the multi-label machine learning experiments and save the results to database. The code’s Javadoc is included. [file 1471-2105-15-150-S2.zip › additional file 2/ml2db/ml2db/doc/index-files/index-19.html]

S-Index


JavaScript is disabled on your browser.


- Overview
- Package
- Class
- Use
- Tree
- Deprecated
- Index
- Help

- Prev Letter
- Next Letter

- Frames
- No Frames

- All Classes

A B C D E F G H I J K L M N O P Q R S T U V W X 


## S

save(TableRow, int) - Method in class main.ml2db.al.database.AlDataSaver


saveArffRecord(Properties) - Method in class main.ml2db.weka.ArffRecordsTable


saveExperimentToDb() - Method in class main.ml2db.mulan.predict.PredictionsSaver


saveRow(Object[]) - Method in class main.ml2db.mulan.learn.crosseval.EvaluationResultsTable
:   Save the evaluation results to table.

saveRow(String, String, int, int, String, String, int) - Method in class main.ml2db.mulan.predict.PredictionsExperimentTable


saveRowPreparedStatement(int, String[], String[], double[], String[]) - Method in class main.ml2db.mulan.predict.PredictionsRecordTable
:   Save using a prepared statement in small batches.

saveStatisticsAsProperties(String, String, String) - Method in class main.ml2db.weka.ArffProps


saveToFileAllDistances() - Method in class main.ml2db.weka.JaccardDistanceStored
:   Generate and print distances for each instance vs each other instance.

saveTrueNegatives() - Method in class main.ml2db.mulan.predict.PredictionsSaver


saveUtilityEntry(int, int, String, double) - Method in class main.ml2db.al.database.AlDataSaver


serialize(MultiLabelLearner) - Method in class main.ml2db.mulan.learn.MulanSerializer


serialize() - Method in class main.ml2db.Serializer


serialize(DistancesCalculator, String) - Static method in class main.ml2db.SerializerHelper


Serializer - Class in main.ml2db
:   Class

Serializer() - Constructor for class main.ml2db.Serializer


SerializerHelper - Class in main.ml2db
:   Class

SerializerHelper() - Constructor for class main.ml2db.SerializerHelper


SerializerTest - Class in test.ml2db.mulan.mulanarff
:   Class

SerializerTest() - Constructor for class test.ml2db.mulan.mulanarff.SerializerTest


serializeUnlabelledPositions(int) - Method in class main.ml2db.al.utility.globaldistance.GlobalDistanceMetric
:   Before serialisation, fill global positions.

setArffRecordId(int) - Method in class main.ml2db.weka.Arff
:   Sets the arff record id and returns it for further use.

setAttributeIndices(String) - Method in class main.ml2db.weka.JaccardDistanceStored


setAttributesNameTypeMap(TreeMap<String, String>) - Method in class main.ml2db.weka.AttributeFactory


setClassAttributes() - Method in class main.ml2db.mulan.mulanarff.IdMultiLabelInstances


setCurrentNeighbourhoodName() - Method in class main.ml2db.al.utility.pickers.byfrequency.NearestNeighboursByFreqUtility


setCvMaxK(int) - Method in class main.ml2db.mulan.classifier.lazy.BRkNNVisible
:   set the maximum number of neighbors to be evaluated via cross-validation

setDataSetManager() - Method in class main.ml2db.mulan.mulanarff.MulanArff


setDataSetManager() - Method in class main.ml2db.weka.Arff


setDebug(boolean) - Method in class main.ml2db.al.ActiveLearning


setDebug(boolean) - Method in class main.ml2db.al.utility.globaldistance.DistancesCalculator


setDebug(boolean) - Method in class main.ml2db.al.utility.globaldistance.DistancesStorage


setDebug(boolean) - Method in class main.ml2db.al.utility.globaldistance.GlobalDistanceMetric


setDebug(boolean) - Method in class main.ml2db.al.utility.pickers.RandomPicker


setDebug(boolean) - Method in class main.ml2db.mulan.learn.evaluation.MicroFMeasurePrinter


setDebugEvaluationResults(boolean) - Method in class main.ml2db.al.ActiveLearning


setDistanceFunction(MultiLabelInstances, NormalizableDistance) - Method in class main.ml2db.al.utility.globaldistance.DistancesCalculator
:   Generates a default Eucidean distance if the distance function is null

setEmptyLabelledSet(boolean) - Method in class main.ml2db.al.utility.globaldistance.GlobalDistanceMetric


setEndTime(String) - Method in class main.ml2db.mulan.learn.evaluation.EvaluatorNoAUC


setEnoughLabelledInstances() - Method in class main.ml2db.al.ActiveLearning


setFeaturesByDescendentFrequency() - Method in class main.ml2db.al.utility.pickers.byfrequency.ByFrequencyPicker


setFeatureToInstanceMap(OneToManyMap<String, String>) - Method in class main.ml2db.al.utility.pickers.RandomPicker


setFileForSerializedModel() - Method in class main.ml2db.Serializer


setFileName(String, int) - Method in class main.ml2db.al.database.AlDataSaver


setFilePath(String) - Method in class main.ml2db.al.utility.globaldistance.DistancesCalculator


setFold(int) - Method in class main.ml2db.mulan.predict.PredictionsSaver


setFrequencyToFeatureMap() - Method in class main.ml2db.al.utility.pickers.byfrequency.ByFrequencyPicker


setInputManager(IdMultiLabelInstances) - Method in class main.ml2db.mulan.learn.crosseval.CrossExperimenter


setInputManager(int, DbManagers) - Method in class main.ml2db.mulan.learn.crosseval.CrossExperimenter


setInputManager(IdMultiLabelInstances) - Method in class main.ml2db.mulan.learn.traintest.TrainTestExperimenter


setInputManager(int, DbManagers) - Method in class main.ml2db.mulan.learn.traintest.TrainTestExperimenter


setInputManager(int, DbManagers, TrainTestExperimenter) - Method in class main.ml2db.mulan.learn.traintest.TrainTestExperimenter


setInstances(IdMultiLabelInstances, IdMultiLabelInstances) - Method in class main.ml2db.al.utility.globaldistance.GlobalDistanceMetric


setInstances(Instances) - Method in class main.ml2db.weka.JaccardDistanceStored


setInstancesNames() - Method in class main.ml2db.weka.DataSetGenerator
:   The instances are all instances with a class + all instances with at
    least one attribute value.

setInvertSelection(boolean) - Method in class main.ml2db.weka.JaccardDistanceStored


setIsNeededBy(Neighbourhood) - Method in class main.ml2db.al.utility.neighbours.Neighbourhood


setkSelectionViaCV(boolean) - Method in class main.ml2db.mulan.classifier.lazy.BRkNNVisible


setLabelled(boolean) - Method in class main.ml2db.al.utility.neighbours.Neighbourhood


setLabInstances(IdMultiLabelInstances) - Method in class main.ml2db.al.ActiveLearning


setLabInstances(IdMultiLabelInstances) - Method in class main.ml2db.al.learning.ExperimentDetails


setLearner(MultiLabelLearner) - Method in class main.ml2db.mulan.learn.MulanLearner


setLogFile(File) - Method in class main.ml2db.al.ActiveLearning


setLogFile() - Method in class main.ml2db.Serializer


setNeedsNominalOrNumeric(String) - Method in class main.ml2db.weka.InstAttValueMap


setNeighboursAndNeedy() - Method in class main.ml2db.al.utility.neighbours.NeighbourhoodNetwork


setNumAttributesWithExamples(MultiLabelInstances) - Method in class main.ml2db.mulan.mulanarff.Statistics


setNumberOfNearestNeighbours(int) - Method in class main.ml2db.al.utility.neighbours.Neighbourhood


setNumLabelsWithExamples(MultiLabelInstances) - Method in class main.ml2db.mulan.mulanarff.Statistics


setOptions(String[]) - Method in class main.ml2db.weka.JaccardDistanceStored


setOutputManager(DbManager, String, Table) - Method in class main.ml2db.al.learning.AlExperimenter


setOutputManager(DbManager, String, Table) - Method in class main.ml2db.mulan.learn.crosseval.CrossExperimenter


setOutputManager(DbManager, String, Table) - Method in class main.ml2db.mulan.learn.traintest.TrainTestExperimenter


setPercentPotentiallyLabelled(double) - Method in class main.ml2db.al.utility.neighbours.Neighbourhood


setStep(int) - Method in class main.ml2db.al.utility.pickers.RandomPicker


setTableName(String) - Method in class test.ml2db.dataharness.DataTableTest


setTest(boolean) - Method in class main.ml2db.al.ActiveLearning


setTest(boolean) - Method in class main.ml2db.al.utility.globaldistance.DistancesStorage


setTest(boolean) - Method in class main.ml2db.al.utility.globaldistance.GlobalPositionStore


setTest(boolean) - Method in class main.ml2db.al.utility.pickers.byfrequency.NearestNeighboursByFreqUtility


setTest(boolean) - Method in class main.ml2db.al.utility.pickers.RandomPicker


setTestArffId(int) - Method in class main.ml2db.mulan.learn.crosseval.CrossExperimenter


setTestArffRecord(int, DbManagers) - Method in class main.ml2db.mulan.learn.traintest.TrainTestInputManager


setTestDataSet(IdMultiLabelInstances) - Method in class main.ml2db.mulan.learn.traintest.TrainTestInputManager


setTrainDataSet(IdMultiLabelInstances) - Method in class main.ml2db.mulan.learn.crosseval.CrossInputManager


setTrainedLearner(MulanLearner) - Method in class main.ml2db.al.ActiveLearning


setTrainedModelFile(String) - Method in class main.ml2db.mulan.learn.traintest.TrainTestExperimenterSerialized


setUnlabelled(IdMultiLabelInstances) - Method in class main.ml2db.al.utility.UtilityMetric


setUnlabInstances(IdMultiLabelInstances) - Method in class main.ml2db.al.ActiveLearning


setUnlabInstances(IdMultiLabelInstances) - Method in class main.ml2db.al.learning.ExperimentDetails


setup() - Method in class main.ml2db.weka.Arff
:   Sets up the data sources, destination etc.

setUp() - Method in class main.ml2db.weka.JaccardDistanceTest


setUp() - Method in class test.ml2db.mulan.learn.CrossEvaluatorTest


setUp() - Method in class test.ml2db.mulan.learn.database.MulanDbWriterTest


setUp() - Method in class test.ml2db.mulan.learn.database.ResultsFormatterTest


setUp() - Method in class test.ml2db.mulan.learn.EvaluationParametersTest


setUp() - Method in class test.ml2db.mulan.learn.EvaluationResultsTableTest


setUp() - Method in class test.ml2db.mulan.learn.ExperimenterTest


setUp() - Method in class test.ml2db.mulan.learn.MulanLearnerTest


setUp() - Method in class test.ml2db.mulan.mulanarff.MulanDataSetDbLoaderTest


setUp() - Method in class test.ml2db.mulan.mulanarff.SerializerTest


setUp() - Method in class test.ml2db.weka.ArffPropsTableTest


setUp() - Method in class test.ml2db.weka.ArffTest


setUp() - Method in class test.ml2db.weka.DataSetCheckerTest


setUp() - Method in class test.ml2db.weka.DataSetGeneratorTest


setUp() - Method in class test.ml2db.weka.DataSetManagerTest


setUp() - Method in class test.ml2db.weka.DataSetWriterTest


setWekaInstance(Instance) - Method in class main.ml2db.mulan.mulanarff.IdInstance


SHAPE\_GREEN\_BOX - Static variable in class main.ml2db.mulan.mulanarff.NeighboursPrinter


SHAPE\_ORANGE\_BOX - Static variable in class main.ml2db.mulan.mulanarff.NeighboursPrinter


SOFTWARE\_PROJECT\_NAME - Static variable in class main.ml2db.ProjectParameters


STANDARD\_BALANCE - Static variable in class main.ml2db.al.utility.globaldistance.GlobalDistanceMetric
:   An equal (50%) weight between distance to labelled and unlabelled
    instances.

start\_graph() - Method in class main.ml2db.graphviz.GraphViz
:   Returns a string that is used to start a graph.

START\_TIMESTAMP - Static variable in class main.ml2db.al.database.AlExperimentsTable
:   time at beginning of computation

startTime() - Method in class main.ml2db.mulan.predict.PredictionsSaver


Statistics - Class in main.ml2db.mulan.mulanarff
:   Class exteded from Mulan
     Class for calculating statistics of a multilabel
    dataset

Statistics() - Constructor for class main.ml2db.mulan.mulanarff.Statistics


STATISTICS\_PATH - Static variable in class main.ml2db.ProjectParameters


statisticsKeys() - Method in class main.ml2db.weka.ArffProps
:   The data statistics properties (number of attributes, classes, instances)
    added after the arff file generation

StatisticsTest - Class in test.ml2db.al
:   Class

StatisticsTest() - Constructor for class test.ml2db.al.StatisticsTest


StatisticsTest - Class in test.ml2db.mulan.mulanarff
:   Class

StatisticsTest() - Constructor for class test.ml2db.mulan.mulanarff.StatisticsTest


statsPropertiesAreCorrect(Properties) - Method in class main.ml2db.weka.ArffProps


STATUS - Static variable in class main.ml2db.mulan.predict.PredictionsRecordTable
:   the prediction status compared with the true label: true positive, false
    positive etc.

STEP\_ID - Static variable in class main.ml2db.al.database.AlDbCreator
:   the table column to store the step identifier (one step is what takes to
    obtain one query instance)

StratifiedFolds - Class in main.ml2db.mulan.learn.crosseval
:   Generate stratified folds with Iterative Stratification

StratifiedFolds(IdMultiLabelInstances, int) - Constructor for class main.ml2db.mulan.learn.crosseval.StratifiedFolds


suite() - Static method in class test.ml2db.alltests.AllActiveLearningTests


suite() - Static method in class test.ml2db.alltests.AllArffTests


suite() - Static method in class test.ml2db.alltests.AllDataTests


suite() - Static method in class test.ml2db.alltests.AllExternalPredictionsEvaluationTests


suite() - Static method in class test.ml2db.alltests.AllGraphVizTests


suite() - Static method in class test.ml2db.alltests.AllMulanArffTests


suite() - Static method in class test.ml2db.alltests.AllMulanLearningTests


suite() - Static method in class test.ml2db.alltests.AllMulanPredictionTests


suite() - Static method in class test.ml2db.alltests.AllMulanTests


suite() - Static method in class test.ml2db.alltests.AllPreliminaryTests


suite() - Static method in class test.ml2db.alltests.AllTests


suite() - Static method in class test.ml2db.alltests.AllTrainTestsTests


SVG - Static variable in class main.ml2db.graphviz.GraphViz

A B C D E F G H I J K L M N O P Q R S T U V W X

- Overview
- Package
- Class
- Use
- Tree
- Deprecated
- Index
- Help

- Prev Letter
- Next Letter

- Frames
- No Frames

- All Classes
